# L-Menthol Attenuates Acetaminophen-Induced Acute Liver Injury Associated with Reduced Oxidative Stress and Ferroptosis-Related Changes

**DOI:** 10.3390/cimb48070655

**Published:** 2026-06-25

**Authors:** Menglong Xu, Yongchao Li, Wenqiang Sun, Haocheng Guan, Tinghui Wu, Shuwei Li

**Affiliations:** 1 State Key Laboratory Incubation Base for Conservation and Utilization of Bio-Resource in the Tarim Basin, Department of Biochemistry and Molecular Biology, College of Life Science and Technology, Tarim University, Alar 843300, China; xumenglong1989@163.com (M.X.); 18850569602@163.com (Y.L.); swq9577@163.com (W.S.); ghc13703476155@163.com (H.G.); 2 School of Medical Technology, Xinjiang Hetian College, Hetian 848000, China

**Keywords:** acetaminophen, liver injury, L-menthol, oxidative stress, ferroptosis, Nrf2

## Abstract

Acetaminophen (APAP) overdose is a major cause of drug-induced liver injury and remains a widely used model of xenobiotic-induced hepatotoxicity. Oxidative stress, mitochondrial dysfunction, and ferroptosis are key events in APAP-mediated liver damage. In this study, we investigated whether L-menthol pretreatment protects against APAP-induced acute liver injury and explored the underlying mechanisms in vivo and in vitro. Male C57BL/6 mice were pretreated with L-menthol (100 mg/kg/day) for 7 days before APAP challenge (300 mg/kg). L-menthol markedly attenuated hepatic necrosis, inflammatory infiltration, and hepatocyte injury, reduced serum alanine aminotransferase (ALT) and aspartate aminotransferase (AST) activities, suppressed IL-1β, IL-6, and TNF-α production, restored hepatic glutathione and superoxide dismutase levels, and decreased malondialdehyde accumulation. Transcriptomic analysis revealed significant enrichment of differentially expressed genes in reactive oxygen species- and ferroptosis-related pathways. In APAP-challenged HepG2 cells, L-menthol improved cell viability, preserved mitochondrial ultrastructure, reduced ferrous iron accumulation, was associated with upregulation of Keap1/Nrf2/HO-1/NQO1 pathway-related proteins, and restored GPX4 expression. Collectively, these findings indicate that L-menthol pretreatment attenuates APAP-induced hepatotoxicity, possibly through enhancement of antioxidant defenses and attenuation of ferroptosis-associated changes, supporting its potential as a preventive hepatoprotective small molecule against xenobiotic-induced liver injury.

## 1. Introduction

Acetaminophen (APAP) is among the most widely consumed analgesics and antipyretics globally, yet its narrow therapeutic index renders overdose the predominant cause of drug-induced liver injury (DILI) in Western countries [1,2]. Mechanistically, excess APAP undergoes cytochrome P450-mediated bioactivation to N-acetyl-p-benzoquinone imine (NAPQI), which depletes hepatic glutathione (GSH) stores and forms covalent protein adducts that precipitate mitochondrial dysfunction, oxidative stress, and hepatocyte necrosis [3,4]. The ensuing release of damage-associated molecular patterns triggers sterile inflammation that further amplifies tissue destruction, and APAP hepatotoxicity can progress rapidly to acute liver failure [5,6]. N-acetylcysteine (NAC) remains the sole approved antidote, acting primarily by replenishing GSH and scavenging reactive oxygen species (ROS) [7]. However, NAC efficacy is constrained by a narrow therapeutic window—optimally within 8 h of ingestion—and potential adverse reactions [8]. These limitations highlight the need for safer, mechanistically distinct hepatoprotective strategies, particularly those derived from natural dietary sources with established safety profiles.

L-Menthol (CAS 2216-51-5), a monocyclic monoterpene alcohol and the principal bioactive constituent of peppermint (Mentha × piperita), is widely used as a food-grade flavoring agent and has well-characterized anti-inflammatory, antioxidant, antibacterial, and antitumor properties [9,10]. Previous studies have suggested that menthol exerts hepatoprotective effects in chemically induced and inflammation-associated liver injury models. Janbaz and Gilani reported that menthol reduced paracetamol- and CCl_4_-induced hepatic damage, as reflected by improved survival and reduced serum liver injury markers [11]. More recently, Matouk et al. showed that menthol attenuated sepsis-induced hepatic injury by reducing oxidative stress, inflammatory mediators, and apoptosis-related signaling, while improving liver regeneration-related responses [12]. However, these studies mainly focused on biochemical, inflammatory, and apoptotic indices, whereas whether L-menthol protects against APAP-induced liver injury through the Keap1/Nrf2/HO-1/NQO1 antioxidant axis and GPX4-associated ferroptosis-related changes remains unclear. Ferroptosis is an iron-dependent and lipid peroxidation-driven form of regulated cell death [13]. Recent reviews have emphasized that Nrf2 is an important regulator of ferroptosis resistance by controlling antioxidant defense, iron homeostasis, lipid peroxidation, and the cysteine/GSH/GPX4 axis [14,15]. In addition, APAP-induced hepatotoxicity has been linked to GSH depletion, mitochondrial oxidative stress, lipid peroxidation, and ferroptosis-related cell death [16,17,18].

Accordingly, the present study investigated the hepatoprotective effects of L-menthol pretreatment in a murine APAP-induced acute liver injury model and in APAP-challenged HepG2 hepatocytes, integrating histological, biochemical, transcriptomic (RNA-seq), and molecular analyses. The investigation aimed to clarify whether the protective effects of L-menthol pretreatment are associated with enhanced antioxidant defense and attenuation of ferroptosis-associated changes.

## 2. Materials and Methods

### 2.1. Reagents and Cell Cultures

L-Menthol (Cat. No. S30540; purity: 98%, BR grade) was purchased from Shanghai Yuanye Biotechnology (Shanghai, China). For in vivo experiments, L-menthol was suspended in 0.5% carboxymethylcellulose sodium (CMC-Na) and freshly prepared before oral gavage. For in vitro experiments, L-menthol was dissolved in dimethyl sulfoxide (DMSO) to prepare a stock solution, stored at −20 °C, and diluted with culture medium before use; the final DMSO concentration did not exceed 0.1%. HepG2 cells (Cat. No. CL-0103) were cultured in Dulbecco’s Modified Eagle Medium (DMEM; Cat. No. PB180120) supplemented with 10% fetal bovine serum (FBS; Cat. No. 164210) and 1% penicillin-streptomycin (Cat. No. PB180121) at 37 °C in a humidified 5% CO_2_ atmosphere. All cell culture reagents were obtained from Wuhan Procell Life Science & Technology (Wuhan, China).

### 2.2. Animal Model and Pretreatment

Thirty specific pathogen-free (SPF) male C57BL/6 mice (6–8 weeks old, 20–22 g) were purchased from Sipeifu Biotechnology Co., Ltd. (Beijing, China). Following a 5-day acclimatization period, mice were randomly assigned to three groups (*n* = 10/group): (1) Normal control (NC): oral gavage of 0.2 mL 0.5% CMC-Na daily for 7 days, followed by intraperitoneal (i.p.) injection of sterile saline; (2) APAP model: oral gavage of 0.2 mL 0.5% CMC-Na daily for 7 days, followed by i.p. injection of APAP (300 mg/kg in sterile saline); (3) L-menthol pretreatment: oral gavage of L-menthol (100 mg/kg in 0.5% CMC-Na) daily for 7 days, followed by i.p. injection of APAP (300 mg/kg). The dose of L-menthol (100 mg/kg/day) was selected based on a previous in vivo study using menthol (PubChem CID: 16666, corresponding to (−)-menthol/L-menthol) at 100 mg/kg by oral gavage in mice [19]. In all groups, APAP or saline was administered 2 h after the final gavage. Mice were fasted for 12 h before APAP administration with ad libitum access to water. Blood samples were collected via retro-orbital puncture 24 h after APAP administration, after which mice were euthanized and liver tissues were harvested immediately.

### 2.3. Liver Histopathology and Immunohistochemical Staining

Fresh liver tissues were rinsed with phosphate-buffered saline (PBS), blotted dry, and weighed. The liver index was calculated as the ratio of liver weight (mg) to body weight (g). For histological analysis, liver samples were fixed in 4% paraformaldehyde for 24 h at room temperature, dehydrated through graded ethanol and xylene, and paraffin-embedded. Sections (6 μm) were stained with hematoxylin and eosin (H&E) and examined by light microscopy (Nikon Instruments Inc., Tokyo, Japan). For immunohistochemistry, paraffin sections were deparaffinized, rehydrated, and incubated with 3% H_2_O_2_ for 10 min to quench endogenous peroxidase. Sections were then incubated with primary antibodies against Ly6G (Cat. No. bs-2576R, Bioss, Beijing, China) and F4/80 (Cat. No. bs-1182R, Bioss, Beijing, China) overnight at 4 °C, followed by a mouse/rabbit IgG immunohistochemical kit (Cat. No. abs996, Absin, Shanghai, China). DAB was used as the chromogen and hematoxylin for counterstaining. For quantification, liver sections from 3 mice per group were analyzed, and 5 randomly selected non-overlapping fields per section were captured and quantified using ImageJ (version 1.53).

### 2.4. Biochemical Assays and Cytokine Measurement in Serum

Whole blood was allowed to clot at 4 °C overnight; serum was obtained by centrifugation at 12,000× *g* for 15 min at 4 °C. Serum alanine aminotransferase (ALT; Cat. No. C009-2-1) and aspartate aminotransferase (AST; Cat. No. C010-2-1) activities were determined using commercial kits (Jiancheng Bioengineering Institute, Nanjing, China) per the manufacturer’s instructions. Serum concentrations of interleukin-1β (IL-1β; Cat. No. ml098416), interleukin-6 (IL-6; Cat. No. ml098430), and tumor necrosis factor-α (TNF-α; Cat. No. ml002095) were determined by enzyme-linked immunosorbent assay (ELISA; Enzyme-Linked Biotechnology, Shanghai, China).

### 2.5. Measurement of Oxidative Stress Parameters in Liver

Liver tissue was homogenized in ice-cold lysis buffer and centrifuged at 8000× *g* for 10 min at 4 °C. Supernatant levels of glutathione (GSH; Cat. No. BC1175), superoxide dismutase (SOD; Cat. No. BC5165), and malondialdehyde (MDA; Cat. No. BC0025) were determined using commercial assay kits (Solarbio, Beijing, China) according to the manufacturer’s instructions.

### 2.6. TUNEL Assay for Apoptosis Detection

Paraffin-embedded liver sections were deparaffinized and permeabilized with proteinase K for 15 min at room temperature. After washing with PBS, sections were incubated with 50 μL TUNEL working solution (Cat. No. C1086, Beyotime, Shanghai, China) for 1 h at 37 °C in the dark. Sections were counterstained with DAPI, mounted, and examined by inverted fluorescence microscopy. For quantification, liver sections from 3 mice per group were analyzed, and 5 randomly selected non-overlapping fields per section were captured and quantified using ImageJ.

### 2.7. Transcriptome Profiling via mRNA Sequencing

Liver tissue samples (*n* = 3 per group) were submitted to Gidio Biology (Guangzhou, China) for mRNA sequencing. Total RNA was extracted using TRIzol (Thermo Fisher Scientific, Waltham, MA, USA) following the manufacturer’s protocol. Total RNA integrity was verified using an Agilent 2100 Bioanalyzer (RIN > 7.0), Agilent Technologies, Santa Clara, CA, USA. Poly(A)-enriched mRNA libraries were constructed and sequenced to generate 150 bp paired-end reads. All samples yielded ≥6 Gb of clean data, with Q30 bases >90% and a clean data ratio >97%. Reads were aligned to the *Mus musculus* GRCm38 reference genome using HISAT2 (v2.0.5). The average mapping rate to the reference genome was >85%, indicating high sequencing quality and alignment efficiency. Differential expression was analyzed using DESeq2 (version 1.38.3) and edgeR (version 3.40.2) with Benjamini–Hochberg correction for multiple testing. Differentially expressed genes (DEGs) were defined as adjusted *p* < 0.05 and |log_2_ (fold change)| > 1. Volcano plots were generated using ggplot2 (version 3.3.6) in R (version 4.2.0). Gene Ontology (GO) and Kyoto Encyclopedia of Genes and Genomes (KEGG) pathway enrichment analyses were performed at *p* < 0.05.

### 2.8. Cell Viability Assay

HepG2 cells were seeded at 3 × 10^4^ cells/well in 96-well plates and allowed to adhere overnight. For cytotoxicity assessment, cells were treated with APAP at concentrations of 0–40 mM for 24 h. For L-menthol safety evaluation, cells were treated with L-menthol (25–200 μM) for 24 h. For protective-effect experiments, cells were pretreated with L-menthol (6.25–100 μM) for 24 h before the addition of APAP (20 mM) for a further 24 h. At the end of each treatment, 10 μL of CCK-8 solution (Cat. No. CA1210, Solarbio, Beijing, China) was added per well, and plates were incubated for 1 h at 37 °C. Absorbance was recorded at 450 nm using a multimode microplate reader (Model K6600A+, Kaiao Technology Development Co., Ltd., Beijing, China).

### 2.9. Analysis of Cellular Markers

Cell culture supernatants were centrifuged at 1000× *g* for 5 min at room temperature. ALT, AST, IL-1β, IL-6, and TNF-α were quantified in the supernatant as described in Section 2.4. Cell lysates were prepared on ice; intracellular GSH, SOD, MDA, and Fe^2+^ (Cat. No. BC5415, Solarbio, Beijing, China) levels were determined using the corresponding commercial kits.

### 2.10. Transmission Electron Microscopy of Mitochondrial Ultrastructure

HepG2 cells were fixed in 2.5% glutaraldehyde in 0.1 M cacodylate buffer for 2 h at 4 °C, post-fixed in 1% osmium tetroxide, and dehydrated through a graded ethanol series (30–100%), with a final acetone exchange. Samples were infiltrated and embedded in Epon resin. Ultrathin sections (~70 nm) were stained with uranyl acetate and lead citrate and examined by transmission electron microscopy (TEM, Hitachi High-Tech, Tokyo, Japan). Mitochondrial morphology was assessed by an observer blinded to the treatment groups.

### 2.11. Western Blot Analysis

Total protein was extracted with RIPA lysis buffer (Cat. No. PC102, Epizyme, Shanghai, China) supplemented with freshly added PMSF (Cat. No. G2008-1ML, Servicebio, Wuhan, China). Protein concentration was quantified by BCA assay (Cat. No. PC0020, Solarbio, Beijing, China). Equal protein loads (30 μg/lane) were separated by 10% SDS-PAGE, transferred to PVDF membranes, and blocked with 5% non-fat dry milk for 1 h at room temperature. Membranes were incubated overnight at 4 °C with the following primary antibodies: Nrf2 (Cat. No. 80593-1-RR, 1:1000), Keap1 (Cat. No. 10503-2-AP, 1:1000), HO-1 (Cat. No. 10701-1-AP, 1:1000), NQO1 (Cat. No. 11451-1-AP, 1:1000), β-actin (Cat. No. 81115-1-RR, 1:5000; all from Proteintech, Wuhan, China), and GPX4 (Cat. No. GB124327, 1:1000, Servicebio, Wuhan, China). After washing, membranes were incubated for 1 h at room temperature with HRP-conjugated secondary antibodies (Goat Anti-Rabbit IgG-HRP, Cat. No. bs-0295G-HRP, 1:5000; Goat Anti-Mouse IgG-HRP, Cat. No. bs-0296G-HRP, 1:5000, Bioss, Beijing, China). Bands were detected using enhanced chemiluminescence (ECL) reagent. Chemiluminescent signals were captured using an imaging system with exposure times adjusted empirically for each membrane to ensure signals remained within the linear detection range, thereby avoiding overexposure. Band intensities were quantified with ImageJ (version 1.53) and normalized to β-actin. All Western blot experiments were performed in three independent biological replicates (*n* = 3).

### 2.12. Statistical Analysis

All data are expressed as the mean ± standard deviation (SD). Statistical comparisons among three groups were performed by one-way analysis of variance (ANOVA) followed by Tukey’s post hoc test for multiple comparisons using GraphPad Prism 9.5 (GraphPad Software, San Diego, CA, USA). A *p* value < 0.05 was considered statistically significant (* *p* < 0.05, ** *p* < 0.01, *** *p* < 0.001).

## 3. Results

### 3.1. L-Menthol Pretreatment Ameliorates APAP-Induced Acute Liver Injury In Vivo

**Figure 1:**
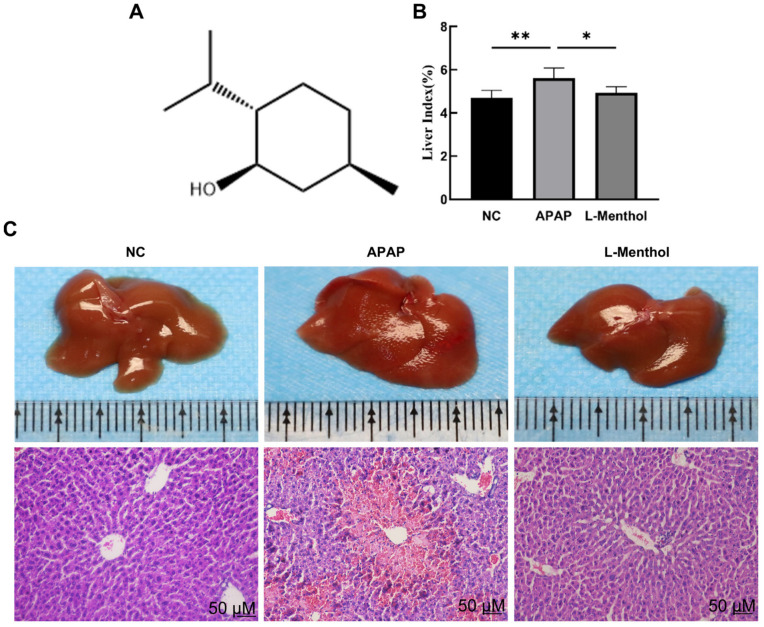
L-Menthol Pretreatment Attenuates APAP-Induced Acute Liver Injury in Mice. (**A**) Chemical structure of L-menthol (CAS: 2216-51-5). (**B**) Liver index of mice in NC, APAP, and L-Menthol pretreatment groups. (**C**) Representative macroscopic photographs (**top**) and H&E histopathological images (**bottom**) of livers from each group. Data are presented as mean ± SD (*n* ≥ 6 per group). * *p* < 0.05, ** *p* < 0.01 vs. APAP group.

As shown in Figure 1, L-menthol pretreatment attenuated APAP-induced liver injury. The liver index was significantly elevated in the APAP group compared with NCs (*p* < 0.01) and was markedly reduced by L-menthol pretreatment (*p* < 0.05) (Figure 1B). Gross examination revealed that APAP-treated livers displayed pronounced surface congestion, granular texture, and darkened parenchyma; in contrast, L-menthol-pretreated livers showed largely restored coloration and surface morphology (Figure 1C). Histopathological evaluation by H&E staining demonstrated extensive hepatocyte ballooning, centrilobular necrosis, widened intercellular spaces, and dense periportal inflammatory infiltration in the APAP group. These pathological changes were substantially attenuated in the L-menthol group, with preservation of near-normal hepatic architecture (Figure 1C).

### 3.2. L-Menthol Pretreatment Normalizes Liver Enzymes, Inflammatory Cytokines, and Oxidative Stress

**Figure 2:**
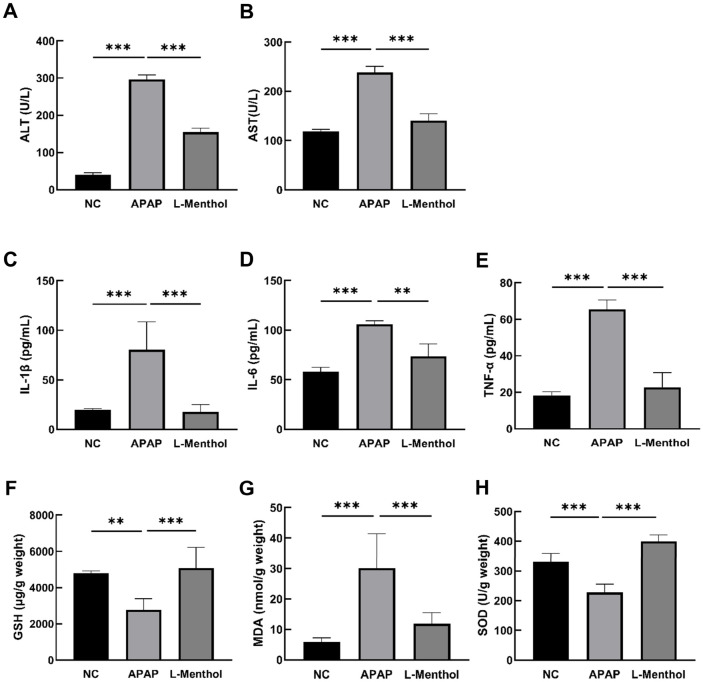
L-Menthol Pretreatment Ameliorates APAP-Induced Liver Injury by Normalizing Liver Enzymes, Inflammatory Cytokines, and Oxidative Stress Markers. (**A**) Serum ALT. (**B**) Serum AST. (**C**) Serum IL-1β. (**D**) Serum IL-6. (**E**) Serum TNF-α. (**F**) Hepatic GSH. (**G**) Hepatic SOD. (**H**) Hepatic MDA. NC: normal control; APAP: acetaminophen model group; L-Menthol: L-Menthol pretreatment group. Data are expressed as mean ± SD. ** *p* < 0.01, *** *p* < 0.001.

Serum ALT and AST activities were significantly elevated in the APAP group relative to NCs (both *p* < 0.001); L-menthol pretreatment significantly attenuated both enzyme activities (both *p* < 0.001 vs. APAP) (Figure 2A,B). Similarly, APAP challenge markedly increased serum levels of pro-inflammatory cytokines IL-1β, IL-6, and TNF-α (all *p* < 0.001 vs. NC), which were substantially reduced by L-menthol pretreatment (all *p* < 0.001 vs. APAP) (Figure 2C–E). Hepatic oxidative stress markers showed that APAP significantly depleted GSH and SOD activity while increasing MDA content (all *p* < 0.001 vs. NC). L-Menthol pretreatment effectively restored GSH and SOD levels and reduced MDA compared with the APAP group (all *p* < 0.001) (Figure 2F–H).

### 3.3. L-Menthol Pretreatment Reduces Hepatic Inflammatory Cell Infiltration

**Figure 3:**
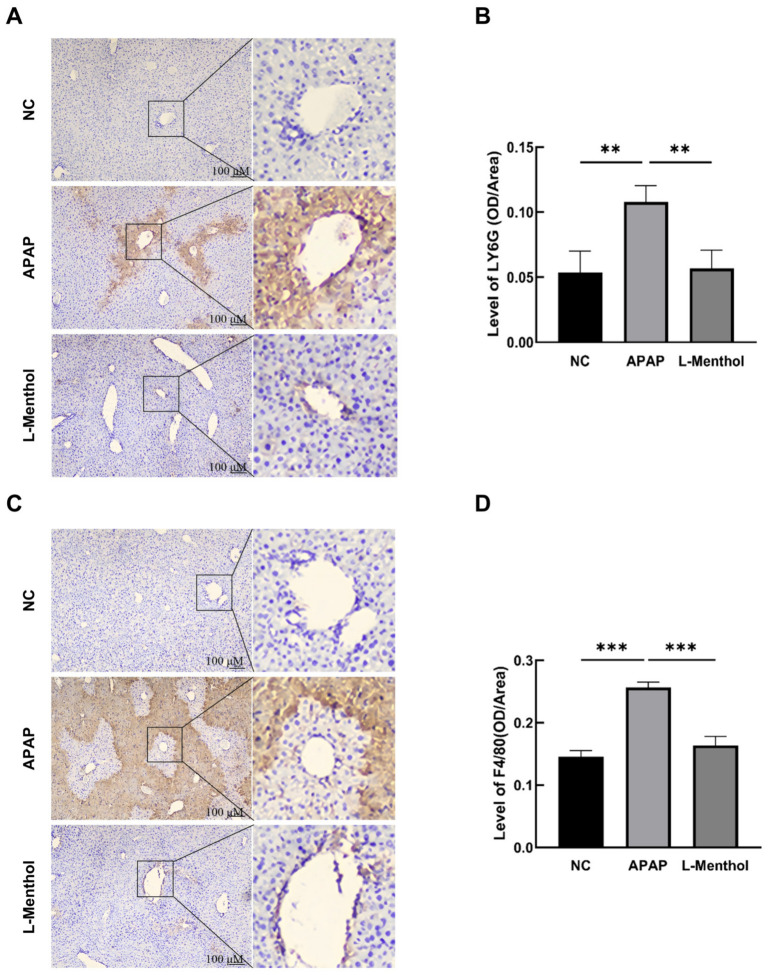
L-Menthol Pretreatment Reduces Hepatic Inflammatory Cell Infiltration in APAP-Induced Liver Injury. (**A**) Representative immunohistochemical images of Ly6G staining. (**B**) Quantification of Ly6G-positive area. (**C**) Representative immunohistochemical images of F4/80 staining. (**D**) Quantification of F4/80-positive area. ** *p* < 0.01, *** *p* < 0.001. Quantification was performed using 5 randomly selected non-overlapping fields per section from 3 mice per group.

Immunohistochemical analysis of Ly6G (neutrophils) and F4/80 (macrophages) revealed that both Ly6G- and F4/80-positive areas were significantly increased in the APAP group compared with NCs (Ly6G: *p* < 0.01; F4/80: *p* < 0.001). L-Menthol pretreatment significantly reduced both Ly6G and F4/80 staining (Ly6G: *p* < 0.01; F4/80: *p* < 0.001 vs. APAP) (Figure 3B,D).

### 3.4. L-Menthol Pretreatment Reduces Hepatocyte Apoptosis

**Figure 4:**
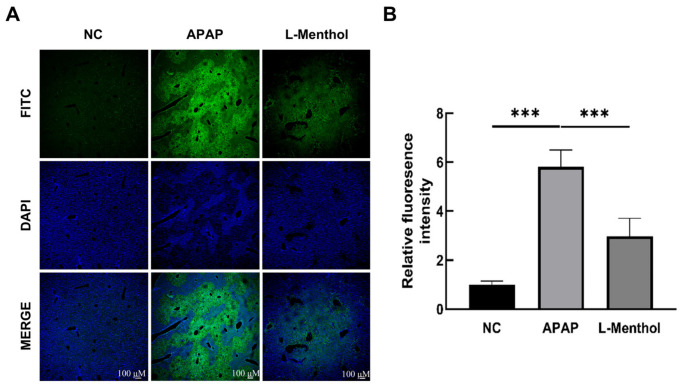
L-Menthol Pretreatment Alleviates APAP-Induced Hepatocyte Apoptosis in Mice. (**A**) Representative TUNEL staining images. Green fluorescence: TUNEL-positive apoptotic cells; blue fluorescence: DAPI-stained nuclei. (**B**) Quantitative analysis of TUNEL-positive cells expressed as relative fluorescence intensity. *** *p* < 0.001. Quantification was performed using 5 randomly selected non-overlapping fields per section from 3 mice per group.

TUNEL staining demonstrated that APAP challenge markedly increased the frequency of TUNEL-positive cells compared with NCs (*p* < 0.001). This apoptotic response was significantly attenuated by L-menthol pretreatment (*p* < 0.001 vs. APAP) (Figure 4A,B).

### 3.5. Transcriptome Sequencing Identifies Ferroptosis and ROS as Key Pathways

**Figure 5:**
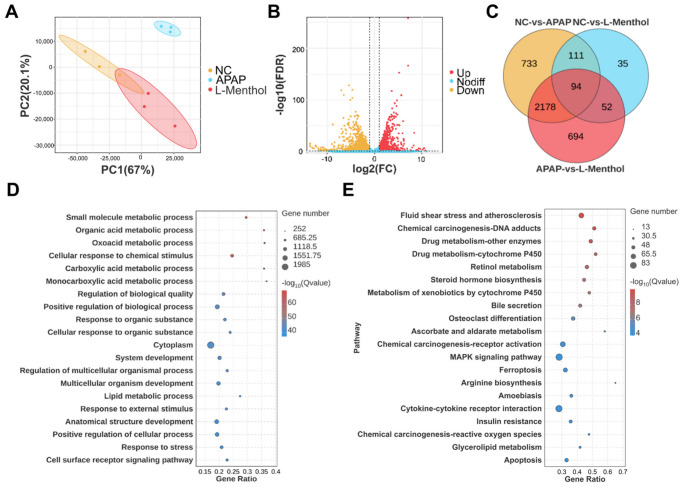
Transcriptomic Analysis Identifies Ferroptosis and ROS as Key Pathways Modulated by L-Menthol. (**A**) PCA plot showing distinct transcriptomic profiles per group. (**B**) Volcano plot identifying 3018 DEGs between the APAP and L-Menthol groups (1156 upregulated, 1862 downregulated). (**C**) Venn diagram highlighting 94 common DEGs. (**D**) GO enrichment analysis. (**E**) KEGG pathway analysis; ferroptosis- and ROS-related pathways are among the top 20 enriched pathways.

To explore the underlying molecular mechanisms, RNA-seq was performed on liver tissue from all three groups (*n* = 3/group). Principal component analysis (PCA) of normalized expression data showed clear separation among NC, APAP, and L-Menthol groups (Figure 5A). A total of 3018 DEGs were identified between the APAP and L-Menthol groups, with 1156 upregulated and 1862 downregulated in L-menthol-treated mice relative to the APAP group (Figure 5B). Venn diagram analysis revealed 94 DEGs shared across all pairwise comparisons (Figure 5C). GO enrichment analysis showed significant enrichment of biological process terms related to small-molecule metabolic processes and molecular function terms related to catalytic activity (Figure 5D). KEGG pathway analysis identified 20 significantly enriched pathways; notably, ferroptosis and chemical carcinogenesis-reactive oxygen species (ROS) pathways ranked among the top enriched terms, along with cytochrome P450 drug metabolism, MAPK, and TNF/NF-κB signaling (Figure 5E).

### 3.6. Establishment of an In Vitro HepG2 Injury Model and Concentration Selection

**Figure 6:**
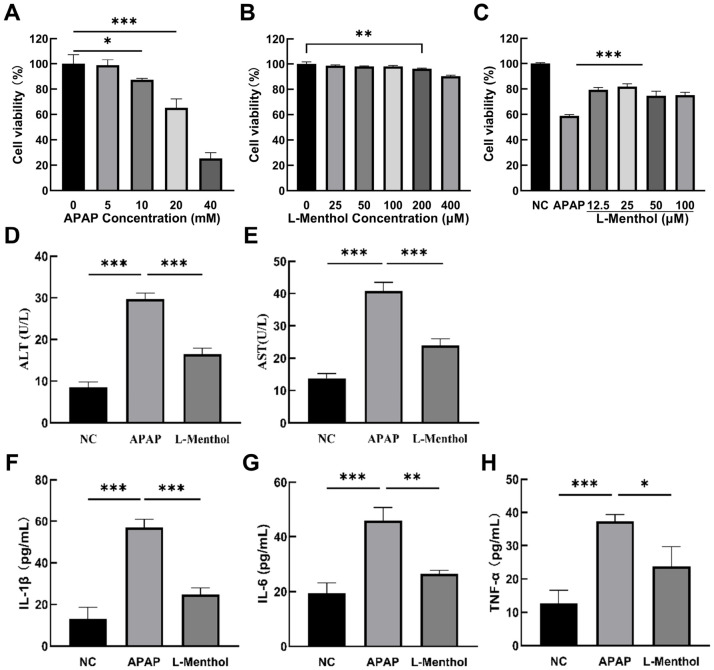
L-Menthol Pretreatment Protects HepG2 Cells from APAP-Induced Hepatocellular Damage and Inflammation. (**A**) Dose-dependent effect of APAP on HepG2 viability. (**B**) Effect of L-menthol on HepG2 viability. (**C**) Protective effect of L-menthol pretreatment on APAP-induced viability loss. (**D**) ALT and (**E**) AST in cell culture supernatant. (**F**) IL-1β, (**G**) IL-6, and (**H**) TNF-α in the supernatant. * *p* < 0.05, ** *p* < 0.01, *** *p* < 0.001.

To investigate the protective mechanisms in vitro, HepG2 cells were treated with varying concentrations of APAP (0–40 mM) for 24 h. Cell viability decreased in a dose-dependent manner, reaching 87.4% at 10 mM (*p* < 0.05), 65.2% at 20 mM (*p* < 0.01), and 25.3% at 40 mM (*p* < 0.01) (Figure 6A). Based on these results, 20 mM APAP was selected for subsequent experiments as it induced substantial but sub-lethal injury. Cell viability assays showed that L-menthol at concentrations of 25–100 μM did not affect baseline viability, whereas 200 μM was cytotoxic (*p* < 0.05) (Figure 6B). Pretreatment with L-menthol (6.25–100 μM) significantly enhanced cell viability in APAP-challenged cells (all *p* < 0.01 vs. APAP), with 25 μM exhibiting the most potent protective effect (Figure 6C). Accordingly, 25 μM L-menthol and 20 mM APAP were used in all subsequent in vitro experiments.

### 3.7. L-Menthol Pretreatment Protects HepG2 Cells from APAP-Induced Hepatocellular Damage and Inflammation

Consistent with the in vivo findings, APAP treatment significantly elevated supernatant ALT and AST in HepG2 cells (both *p* < 0.001 vs. NC), which were markedly reduced by L-menthol pretreatment (both *p* < 0.001 vs. APAP) (Figure 6D,E). Similarly, APAP challenge significantly increased supernatant levels of IL-1β, IL-6, and TNF-α (all *p* < 0.001 vs. NC), and these inflammatory mediators were significantly decreased by L-menthol pretreatment (IL-1β: *p* < 0.001; IL-6: *p* < 0.01; TNF-α: *p* < 0.05 vs. APAP) (Figure 6F–H).

### 3.8. L-Menthol Pretreatment Alleviates Oxidative Stress and Preserves Mitochondrial Morphology in HepG2 Cells

**Figure 7:**
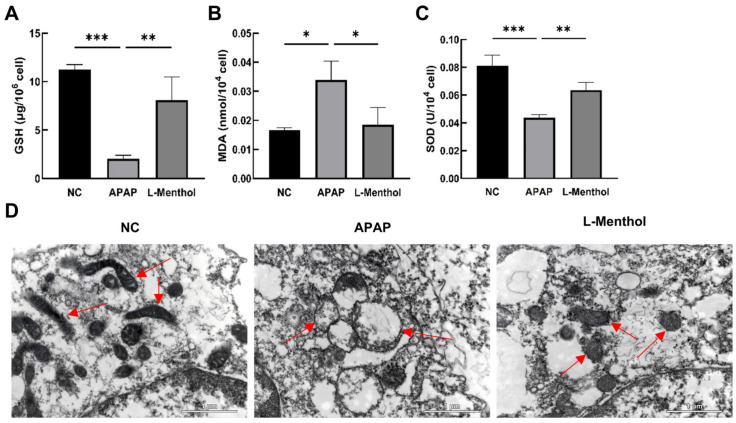
L-Menthol Pretreatment Alleviates Oxidative Stress and Protects Mitochondrial Architecture in APAP-Exposed HepG2 Cells. (**A**) Intracellular GSH. (**B**) MDA. (**C**) SOD in cell lysates. (**D**) Representative TEM images of mitochondria at 30,000× magnification; red arrows indicate representative mitochondria. Scale bar = 1 μm. * *p* < 0.05, ** *p* < 0.01, *** *p* < 0.001.

Intracellular oxidative stress markers were measured in HepG2 cell lysates. APAP significantly reduced GSH content and SOD activity (both *p* < 0.001 vs. NC) while elevating MDA levels (*p* < 0.05 vs. NC). L-Menthol pretreatment effectively restored GSH and SOD (both *p* < 0.01) and reduced MDA (*p* < 0.05) compared with the APAP group (Figure 7A–C). TEM analysis at 30,000× magnification revealed that APAP treatment induced mitochondrial swelling, rounding, cristae fragmentation, and loss of inner membrane integrity, contrasting with the elongated, cristae-dense mitochondria observed in NC cells. Notably, L-menthol-pretreated cells exhibited partially restored tubular morphology with increased cristae density (Figure 7D).

### 3.9. L-Menthol Pretreatment Is Associated with Upregulation of Keap1/Nrf2/HO-1/NQO1 Pathway-Related Proteins

**Figure 8:**
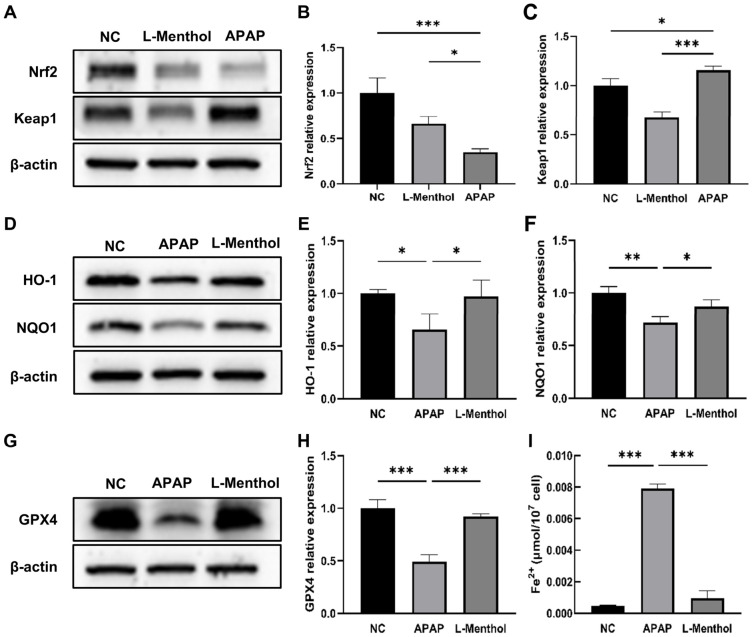
L-Menthol Pretreatment is Associated with Upregulation of Nrf2/HO-1/NQO1 Pathway-Related Proteins and Attenuation of Ferroptosis-Associated Changes in APAP-Exposed HepG2 Cells. (**A**) Western blot of Nrf2 and Keap1. (**B**) Quantification of Nrf2/β-actin. (**C**) Quantification of Keap1/β-actin. (**D**) Western blot of HO-1 and NQO1. (**E**) Quantification of HO-1/β-actin. (**F**) Quantification of NQO1/β-actin. (**G**) Western blot of GPX4. (**H**) Quantification of GPX4/β-actin. (**I**) Intracellular Fe^2+^ levels. Data are representative of three independent biological replicates (*n* = 3). * *p* < 0.05, ** *p* < 0.01, *** *p* < 0.001.

Western blot analysis was performed to evaluate the effects of L-menthol on Keap1/Nrf2/HO-1/NQO1 pathway-related protein expression in HepG2 cells. APAP significantly downregulated Nrf2 (*p* < 0.001 vs. NC) and upregulated Keap1 (*p* < 0.05 vs. NC); L-menthol pretreatment significantly reversed these changes (Nrf2: *p* < 0.05; Keap1: *p* < 0.01 vs. APAP) (Figure 8A–C). Moreover, APAP reduced the protein expression of downstream effectors HO-1 (*p* < 0.05 vs. NC) and NQO1 (*p* < 0.01 vs. NC), both of which were significantly restored by L-menthol pretreatment (both *p* < 0.05 vs. APAP) (Figure 8D–F).

### 3.10. L-Menthol Pretreatment Attenuates Ferroptosis-Associated Changes with Restoration of GPX4 Expression

Given the RNA-seq findings indicating ferroptosis pathway enrichment, we assessed ferroptosis-associated markers in HepG2 cells. APAP treatment significantly increased intracellular Fe^2+^ levels (*p* < 0.05 vs. NC) and significantly decreased GPX4 protein expression (*p* < 0.001 vs. NC). L-Menthol pretreatment significantly reduced Fe^2+^ accumulation and restored GPX4 expression compared with the APAP group (*p* < 0.001 vs. APAP) (Figure 8G–I).

## 4. Discussion

APAP-induced liver injury remains a significant clinical challenge, and the identification of food-derived bioactive compounds with hepatoprotective potential is of considerable translational interest. In this study, we demonstrated that oral pretreatment with L-menthol significantly attenuated APAP-induced acute liver injury in mice, accompanied by reduced oxidative stress, inflammation, apoptosis, and ferroptosis-associated changes. Integrating in vivo, in vitro, and transcriptomic evidence, our data suggest that the Keap1/Nrf2/HO-1/NQO1 antioxidant axis and GPX4-associated ferroptosis-related changes appear to be involved in the protective effects of L-menthol pretreatment. These findings extend the previously reported hepatoprotective effects of menthol in liver injury models [11,12] by suggesting a potential mechanistic link involving antioxidant defense and ferroptosis-associated pathways, which have been implicated in APAP-induced hepatotoxicity [16,17,18].

In our murine model, L-menthol pretreatment reduced the liver index, attenuated centrilobular necrosis and inflammatory infiltration on histology, and lowered serum ALT and AST, confirming hepatoprotection consistent with earlier menthol studies [11]. Notably, L-menthol concurrently suppressed serum IL-1β, IL-6, and TNF-α and reduced hepatic Ly6G^+^ neutrophil and F4/80^+^ macrophage accumulation. The innate inflammatory response is a well-established amplifier of APAP-induced hepatocellular damage [3,6]. Although L-menthol is a recognized agonist of the transient receptor potential melastatin 8 (TRPM8) channel, which has been implicated in macrophage-mediated cytokine regulation [20], the contribution of TRPM8 signaling to the hepatoprotective effects observed here was not directly assessed and warrants future investigation.

An important observation is that L-menthol pretreatment was associated with upregulation of Keap1/Nrf2/HO-1/NQO1 pathway-related proteins in APAP-challenged HepG2 cells. Western blot analysis showed that L-menthol decreased Keap1 and increased Nrf2 protein levels, with concomitant upregulation of the downstream effectors HO-1 and NQO1. Consistent with these protein-level changes, L-menthol pretreatment restored intracellular GSH and SOD activity and reduced MDA levels both in vivo and in vitro. These observations are consistent with the hepatoprotective mechanisms reported for other natural Nrf2 activators in APAP models, including astaxanthin [18], kaempferol [13], and urolithin A [7]. However, L-menthol is distinguished from these compounds by its established use as a food-grade ingredient and its widespread dietary exposure [9], features that support further investigation of its hepatoprotective potential. In the present study, changes in Nrf2 signaling were inferred from total protein expression of Nrf2 and its downstream effectors; however, Nrf2 nuclear translocation and pathway causality were not directly assessed. Whether L-menthol directly modifies Keap1 cysteine residues or acts indirectly through upstream signals such as TRPM8-dependent calcium influx or ROS-mediated Keap1 oxidation remains to be determined.

The modulation of ferroptosis-associated markers by L-menthol is an important observation of this study. Our RNA-seq analysis identified ferroptosis among the enriched KEGG pathways in the APAP vs. L-menthol comparison, suggesting that ferroptosis-related processes appear to be involved in the protective effects of L-menthol. This pathway-level enrichment is consistent with previous studies showing that APAP-induced hepatotoxicity involves oxidative stress, GSH depletion, iron-related dysregulation, lipid peroxidation, and GPX4-associated ferroptosis-related injury [13,16,17,18]. At the protein level, L-menthol reversed the APAP-induced decrease in GPX4 expression and attenuated intracellular Fe^2+^ accumulation. GPX4 is the principal enzymatic defense against ferroptotic lipid peroxidation, and its insufficiency—driven by GSH depletion—has been increasingly recognized as a contributor to APAP hepatotoxicity [7,13,18]. The ability of L-menthol to restore both GSH and GPX4 protein levels suggests that antioxidant defense and GPX4-associated lipid peroxide detoxification may jointly contribute to its protective effects. TEM analysis further revealed that L-menthol partially preserved mitochondrial morphology in APAP-challenged HepG2 cells, reducing the swelling, cristae fragmentation, and inner membrane disruption that characterize APAP-induced mitochondrial damage [6]. The preservation of mitochondrial ultrastructure is therefore likely associated with the combined antioxidant effects and attenuation of ferroptosis-associated changes, rather than representing a primary mechanism.

Several limitations should be noted. The pretreatment design limits the findings to a protective rather than therapeutic context, fasting before APAP administration may have influenced hepatic redox balance as a confounding variable, and the HepG2 model has limited CYP2E1 activity; additionally, histological and TEM analyses were descriptive without blinded scoring or quantitative mitochondrial damage assessment, and future studies should incorporate lipid ROS detection, assessment of ACSL4 and SLC7A11 expression, ferroptosis inhibitor rescue experiments, and direct assessment of Nrf2 nuclear translocation to further validate the proposed mechanisms.

## 5. Conclusions

In conclusion, this study demonstrates that L-menthol pretreatment protects against APAP-induced acute liver injury. This protective effect was associated with enhanced antioxidant defense, reduced Fe^2+^ accumulation, restoration of GPX4 expression, and preservation of mitochondrial ultrastructure. These findings suggest that the Keap1/Nrf2/HO-1/NQO1 antioxidant axis and ferroptosis-associated changes appear to be involved in the hepatoprotective effect of L-menthol pretreatment. However, whether L-menthol has therapeutic efficacy after APAP-induced liver injury requires further investigation.

## Data Availability

Data will be made available upon request.

## Abbreviations

The following abbreviations are used in this manuscript:

APAP	Acetaminophen
ALT	Alanine Aminotransferase
AST	Aspartate Aminotransferase
CMC-Na	Carboxymethylcellulose Sodium
DILI	Drug-Induced Liver Injury
DMSO	Dimethyl Sulfoxide
GPX4	Glutathione Peroxidase 4
GSH	Glutathione
HO-1	Heme Oxygenase-1
IL-1β	Interleukin-1β
IL-6	Interleukin-6
TNF-α	Tumor Necrosis Factor-α
KEGG	Kyoto Encyclopedia of Genes and Genomes
MDA	Malondialdehyde
NAC	N-Acetylcysteine
NAPQI	N-Acetyl-p-benzoquinone Imine
NQO1	NAD(P)H Quinone Dehydrogenase 1
Nrf2	Nuclear Factor Erythroid 2-related Factor 2
ROS	Reactive Oxygen Species
SOD	Superoxide Dismutase
SPF	Specific Pathogen-Free
TEM	Transmission Electron Microscopy
TUNEL	Terminal Deoxynucleotidyl Transferase dUTP Nick End Labeling
